# Study of the reparative effects of menstrual-derived stem cells on premature ovarian failure in mice

**DOI:** 10.1186/s13287-016-0458-1

**Published:** 2017-01-23

**Authors:** Zhen Wang, Yueling Wang, Ting Yang, Jing Li, Xinyuan Yang

**Affiliations:** 1 Department of Gynecology and Obstetrics, First Affiliated Hospital, Xi’an Jiaotong University, Xi’an, 710061 People’s Republic of China; 2 Center for Translational Medicine, First Affiliated Hospital of Xi’an Jiaotong University, Xi’an, 710061 People’s Republic of China

**Keywords:** Premature ovarian failure, Menstrual-derived stem cells, Paracrine, Fibroblast growth factor 2

## Abstract

**Background:**

Young female patients who receive chemotherapy frequently face premature ovarian failure (POF). The therapeutic potential of stem cells in these patients has been explored in stem cells derived from different sources. However, many of these types of stem cells are either difficult to obtain or obtaining them involves invasive procedures. Here, we show that menstrual-derived stem cells (MenSCs) are easy to access and exhibit mesenchymal stem cell-like properties. MenSCs are therefore a novel source of stem cells that can be used for tissue repair. The aim of this study was to explore the reparative capacity and the mechanism underlying the activities of MenSCs.

**Methods:**

POF mouse models were established by 7 consecutive days of intraperitoneal injection of cisplatin, and then MenSCs or MenSC-derived conditioned media (CM) were infused via the tail vein. The ovaries were excised after either 7 or 21 days of treatment and the follicles were counted and categorized. Apoptosis of granulosa cells was observed by terminal deoxynucleotidyl transferase mediated dUTP nick end labelling staining. Ovarian function was evaluated by monitoring serum sex hormone levels. Furthermore, MenSC tracking, Q-PCR, and small interfering RNA transfection were used to reveal the inner mechanism of repair.

**Results:**

MenSC transplantation could improve the ovarian microenvironment by reducing apoptosis in granulosa cells and the fibrosis of ovarian interstitium, which contributes to increase the follicular numbers and return sex hormone levels to normal values. Meanwhile, the transplanted MenSCs directively migrate to ovarian interstitium to play a role in repair rather than differentiate to oocytes directly. Additionally, MenSCs and CM derived from these cells exerted protective effects on damaged ovaries partially by secreting FGF2.

**Conclusion:**

MenSCs repair ovarian injury, improve ovarian function, and stimulate regeneration, suggesting that transplantation of MenSCs may provide an effective and novel method for treating POF.

## Background

Chemotherapies, which are often used in conjunction with surgery to treat cancer, are unquestionably beneficial therapeutic agents. However, the side effects caused by chemotherapy include damage to the ovaries in female patients, and this effect cannot be ignored because it frequently leads to premature ovarian failure/insufficiency (POF/POI) or menopause as a direct result of its toxic effects on follicles [1–3]. Chemotherapy can even lead to small follicle depletion and a complete loss of oocytes. However, there is currently no clear standard definition for chemotherapy-induced ovarian failure, which is characterized by irreversible amenorrhea in the presence of a negative pregnancy test and follicle-stimulating hormone (FSH) levels higher than 40 mIU/ml after chemotherapy. A large number of female patients suffer adverse consequences following chemotherapy. These can include hot flushes, cardiovascular or neurological system disease, osteoporosis, and sexual dysfunction. These symptoms are dose dependent and cause a host of serious social and psychological problems. Hormone replacement therapy (HRT) has been used to treat POF/POI. However, HRT both increases the risk for recurrence of cancer [4, 5] and fails to solve the fundamental problem. In recent years, interest has rapidly grown in studies exploring the therapeutic potential of stem cells because of their potential to differentiate into various specialized cell types. This characteristic has been observed in stem cells derived from a number of different sources, including bone marrow [6–8], amniotic fluid [9], and adipose tissue [10, 11], and all of these cell types have been shown to have therapeutic effects on long-term infertility and ovarian damage. However, many of the suitable cell types currently identified for human use are either difficult to obtain or obtaining them involves invasive procedures.

Menstrual-derived stem cells (MenSCs) display mesenchymal stem cell (MSC)-like properties, including self-renewal, high rates of proliferation, and multi-differentiation capacity [12]. MenSCs can grow and adhere in vitro and have been shown to be positive for MSC markers [13]. Under specific conditions, MenSCs also undergo multipotent differentiation into various functional cell types, including adipocytes [14], myocytes [15], osteocytes [16], cardiomyocytes [17], neurocytes [18, 19], endothelial cells [20], pancreatic cells [21], and hepatocytes [22–25]. In autologous cell repair and regeneration, MenSCs present major advantages over other sources of MSCs, including ease of access and the ability to achieve repeated sampling in a non-invasive manner. Significantly, MenSCs exhibit low immunogenicity and possess immunoregulatory functions which make them safe in xenogenous transplantation [26]. Hence, MenSCs are an ideal cell type for use in treatments for tissue damage. The efficacy of MenSCs has been demonstrated in several clinical trials, including trials evaluating MenSCs as a treatment for myocardial infarction [27], neuron system diseases [28, 29], diabetes mellitus [30, 31], and multiple sclerosis [32]. Because of their demonstrated differentiation capacity and plasticity, we hypothesized that MenSCs may also be useful as an alternative treatment for POF/POI.

Therefore, in this study, we injected MenSCs via the tail vein into chemotherapy-induced POF mice and then measured their restorative effect on ovarian function. In addition, we further studied the mechanisms underlying MenSC-mediated repairs.

## Methods

### Isolation and culture of cells

The collection of the samples used for research purposes in this study was approved by the Ethical Committee of The First Affiliated Hospital of Xi’an Jiantong University, and written informed consent was obtained from each donor. Menstrual blood (approximately 10 ml each) was collected from six healthy women (25–30 years old) on the first day of menstruation using a menstrual cup (Green Donna, GuangzhouMeiFanle Rubber Products, China). The samples were then transferred into a 50 ml centrifuge tube containing 10 ml of phosphate-buffered saline (PBS), penicillin (100 U/ml), streptomycin (100 mg/ml), 0.25 mg/ml amphotericin B, and 2 mM ethylenediaminetetraacetic acid (EDTA) (Gibco, Grand Island, NY, USA). Menstrual blood mononuclear cells were separated using Ficoll-Paque Plus (GE Healthcare, Amersham, UK) according to the manufacturer’s instructions. The cells were suspended in a T25 flask (Corning, New York, USA) containing DMEM/F12 supplemented with 10% foetal bovine serum (FBS) (SiJiqing, China), streptomycin (100 mg/ml), and penicillin (100 U/ml) and then cultured in a humidified incubator at 37 °C in 5% CO_2_. The cell culture medium was changed every 3 days. When the cells reached 90% confluence, they were detached using 0.25% trypsin–EDTA and passaged at a ratio of 1:3.

### MTT assay to evaluate cell proliferation

Cells at passages P3, P10, and P15 were seeded at 2.0 × 10^3^ cells/well in 96-well plates (Corning) and cultured in DMEM/F12 supplemented with 10% FBS, streptomycin (100 mg/ml), and penicillin (100 U/ml). On each of the following 7 days, MTT (5 mg/ml; Sigma-Aldrich, St Louis, MO, USA) was added to the cell medium at the same time point, and the cells were then incubated at 37 °C for an additional 4 h. A 150-μl volume of dimethylsulfoxide (DMSO; Sigma-Aldrich) was then added to each well to terminate the reaction, and the blue–violet precipitate was lysed for 15 min while shaking the solution. Absorbance values were determined at 490 nm using an ELISA reader (Model 680; Bio-Rad, Hercules, CA, USA). The experiment was performed in triplicate.

### Colony-forming assay

Freshly sorted cells were seeded at a very low seeding density (150 cells/cm^2^) in fibronectin-coated (10 μg/ml) 60-mm cell culture dishes and cultured in stromal medium containing DMEM/F12, 10% FBS, 2 mM glutamine, and 0.5 mg/ml primocin at 37 °C in 5% CO_2_. The medium was changed every 6–7 days. After 14 days, the cells were fixed in methanol and stained using Giemsa. Only colonies containing 50 or more cells were defined as colony-forming units (CFU). The number of colonies was counted according to this definition.

### Flow cytometry analysis

To analyse surface marker expression using flow cytometry, adherent cells (1 × 10^6^) were harvested in 0.02% EDTA, washed twice with PBS, and disaggregated into single cell suspensions by pipetting. The cells were incubated with 10 μl of the following antibodies: fluorescein isothiocyanate (FITC)-conjugated mouse monoclonal antibodies against CD34, CD44, CD45, CD73, and CD90, and phycoerythrin (PE)-conjugated mouse monoclonal antibodies against CD49, CD133, and CD146 (Biosciences, San Jose, CA, USA) at 4 °C for 30 min. The cells were then washed twice with PBS, resuspended in 0.4 ml of PBS, and immediately analysed using a FACS Calibur flow cytometer (Becton Dickinson, CA, USA). Cell Quest software was used for the data analysis.

### Cytogenetic analysis

We used cytogenetic analyses to determine the karyotypes of MenSCs at P25. After the MenSCs were treated with Colcemid, they were harvested using centrifugation and washed twice with PBS. Then, 75 mM KCl was added to the resuspended cells, and the mixture was incubated for 20 min at 37 °C. To this mixture, we added 7 drops of chilled methanol-acetic acid fixative. The mixture was then centrifuged for 10 min at 4 °C. The cell suspension was dropped onto a pre-cooled slide and allowed to air dry. The slides were then observed under a microscope.

### In-vitro differentiation

Cells were differentiated into adipogenic and osteogenic lineages to assess their differentiation capacities in vitro*.*

To achieve adipogenic differentiation, cells were seeded at a density of 1 × 10^4^ cells/cm^2^. After the cells reached 80% confluence, they were incubated in adipogenic differentiation medium for 3 weeks. The cells were then fixed with 4% paraformaldehyde (Sigma-Aldrich) and stained with Oil Red O (Sigma-Aldrich). The differentiation medium was changed every 3 days. The adipogenic medium contained DMEM/F12, 10% FBS, 10^−6^ mol/L dexamethasone, 0.5 mmol/L isobutyl methylxanthine, 10 μg/ml insulin, and 200 μmol/L indomethacin (Sigma-Aldrich).

To achieve osteogenic differentiation, the cells were seeded at a density of 2 × 10^3^ cells/cm^2^. After 24 h, the medium was replaced with osteogenic differentiation medium, and the cells were induced for 2 weeks. The cells were then fixed, and mineral deposition was visualized using Alizarin Red (Sigma Aldrich). The osteogenic medium contained DMEM/F12, 10% FBS, 10^−8^ mol/L dexamethasone, 10 mmol/L β-glycerol phosphoric acid, and 50 μmol/L ascorbic acid (Sigma-Aldrich).

At each experimental endpoint, the cell types of the differentiated cells were identified using RT-PCR analysis. The specific primers used for these experiments are presented in Table 1.

**Table 1:** Primer list

Gene	Forward sequence(5′ → 3′)	Reverse sequence (5′ → 3′)
*SPARC*	TACATCGCCCTGGATGAGTG	CACCTTGTCTCCAGGCAGAAC
*PPARγ*	GGCCAAGGCTTCATGACAA	AATGGGCTTCACATTCAGCAA
*Runx2*	TCCACACCATTAGGGACCATC	TGCTAATGCTTCGTGTTTCCA
*FabP4*	AGAGGATGATAAACTGGTGGTG	CGAACTTCAGTCCAGGTCAA
*VEGF*	CCTTGCTGCTCTACCTCC	AAATGCTTTCTCCGCTCT
*FGF2*	CTAACCGTTACCTGGCTATG	TTATACTGCCCAGTTCGTTT
*IGF-1*	AGGAAGTACATTTGAAGAACGCAAGT	CCTGCGGTGGCATGTCA
*HGF*	GATGTCCACGGAAGAGGAGA	GAGTCACCTTCCCTCGATGA
*G-CSF*	GACCCATGGCTGGACCT	ATGGGGAGGGCTTGGCT
*GAPDH*	GCACCGTCAAGGCTGAGAAC	TGGTGAAGACGCCAGTGGA

### Reverse transcription and real-time qPCR

Total RNA was extracted from the cells using a RNA Fast 200 Kit (Aidlab Biotechnology, China) according to a standard protocol. One microgram of total RNA from each sample was used as the template for the reverse transcription reaction using a RevertAid First Strand cDNA Synthesis Kit (Fermentas, USA). Real-time qPCR was then performed using the cDNA with a SYBR Premix Ex Taq II (Takara, China) and a CFX-96 Real-time PCR Detection System (Bio-Rad, USA). The amplification reaction was performed using 40 cycles of the following conditions: denaturation at 95 °C for 5 s and annealing at 60 °C for 30 s. All gene expression levels were normalized to the level of the internal standard control, Gapdh, and analysed using the 2^−ΔΔCt^ method. The specific primers used in these experiments are presented in Table 1.

### Experimental animals and animal model establishment

Female C57BL/6 mice aged 7–8 weeks were purchased from Xi’an Jiaotong University Animal Laboratory. The experimental protocol was approved by the Ethical Committee and the Institutional Animal Care and Use Committee of Xi’an Jiaotong University. The body weight of each mouse varied from 16 to 18 g and was recorded every day. Vaginal smears were obtained daily. The normal oestrous cycle in mice consists of the following four sequential stages: pro-oestrus, oestrus, metoestrus, and dioestrus. These stages were determined based on the presence or absence of leukocytes, cornified epithelium, and nucleated epithelial cells [33, 34]. Additionally, only mice that went through at least two consecutive normal oestrous cycles were included in the experiments.

Mice were intraperitoneally injected with cisplatin (CDDP; 2 mg/kg) for 7 consecutive days to create the POF model.

### MenSC transplantation

The mice were randomly divided into the following three groups: control group (normal mice without any treatment, *n* = 10), POF group (POF mice, *n* = 20), and MenSC-treated group (POF mice that were injected with 200-μl cell suspensions containing 2 × 10^6^ MenSCs on days 1 and 3 of the experiment, *n* = 20). At 7 and 21 days after treatment was begun, the animals were sacrificed, and serum samples and ovaries were collected for subsequent experiments.

### Tracking GFP-labelled transplanted MenSCs

MenSCs were infected with green fluorescence protein (GFP)-expressing lentiviral vectors (Genechem, China). The GFP-expressing MenSCs were cultured in DMEM/F12 supplemented with 10% FBS (SiJiqing). Before cell transplantation, GFP expression was verified in the MenSCs using a fluorescence microscope. The cells were trypsinized and washed twice with PBS, and the resulting cell pellet was suspended in PBS to achieve a final density of 5 × 10^6^ cells/100 μl. Using a Hamilton syringe, a total of 1 × 10^7^ GFP-labelled MenSCs in 200 μl of PBS were evenly injected into the tail veins of POF mice. At 7 and 21 days after injection, the mice were sacrificed, and frozen sections were made from the ovaries. These were used to investigate the morphologies and locations of GFP-MenSCs under a fluorescence microscope (Olympus, Japan). Nuclei were stained with DAPI.

### Ria method of measuring serum hormone

To analyse ovarian function, collected serum samples were used to measure hormone levels. We measured serum oestradiol (E2) and FSH levels using a [^125^I]Oestradiol Radioimmunoassay Kit (JiuDing, China) and a [^125^I]Human FSH Radioimmunoassay Kit (JiuDing, China) according to a standard protocol.

### Ovarian follicle counts and morphologic analysis

To analyse ovarian morphology, ovaries were collected from the three groups at 7 and 21 days after MenSC transplantation and fixed in 4% paraformaldehyde for 12–16 h. After the ovaries were fixed, they were dehydrated, paraffin-embedded, serially sectioned at 5 μm thick, and mounted on glass microscope slides. Routine haematoxylin and eosin (H&E) staining was performed for histologic examinations, which were analysed under light microscopy. Follicles were categorized and counted in every fifth section through the ovary. Follicles were classified as follows: a primordial follicle was an oocyte that was surrounded by a single layer of squamous granulosa cells; a primary follicle was an intact, enlarged oocyte with a visible nucleus and one layer of cuboidal granulosa cells; a secondary follicle possessed two or three layers of cuboidal granulosa cells without antral space; early antral follicles contained emerging antral spaces; and pre-ovulatory follicles, the largest of the follicular types, possessed a defined cumulus granulosa cell layer [35].

### Apoptosis assay

To detect apoptosis in the collected ovaries, terminal deoxynucleotidyl transferase mediated dUTP nick end labelling (TUNEL) staining kits (Roche Applied Science, USA) were used according to the manufacturer’s instructions. The nuclei were counterstained using DAPI. Images were collected under a fluorescence microscope (Olympus). The percentage of TUNEL-positive cells was determined by counting five random fields from each sample. The results are expressed as the percentages of apoptotic cells in each section.

### Conditioned medium administration in vivo

MenSCs at 80% confluence were switched to serum-free DMEM/F12 and cultured for an additional 48 h. The culture media were then collected as conditioned media (CM) and concentrated 10 times using ultrafiltration centrifuge tubes (molecular weight cut-off value: 3 kDa; Millipore, USA).

Female C57BL/6 mice were sorted into the three following groups: control group (normal animals without any treatment, *n* = 10), POF group (POF model, approximately 200 μl of control DMEM/F12 was injected through the tail vein, *n* = 20), and CM-treated group (POF model, approximately 200 μl of concentrated CM was injected through the tail vein, *n* = 20). The mice were sacrificed 7 days after treatment. The ovaries of the mice were removed for H&E staining or TUNEL assays, and serum samples were collected to measure sex hormone levels.

### RNA interference

Fibroblast growth factor 2 (FGF2) small interfering RNAs (siRNAs) and negative transfection control siRNAs (NTC) were purchased from GenePharma (China). Cells were transfected with double-stranded siRNA oligonucleotides specific for FGF2 (sense sequence: 5′-GGGCAGUAUAAACUUGGAUTT-3′, anti-sense sequence: 5′-AUCCAAGUUUAUACUGCCCTT-3′). MenSCs were incubated with 50 nM siRNA for 6 h. Lipofectamine 2000 reagent (Invitrogen, USA) was used as the transfection reagent. The cells were then switched to fresh DMEM/F12 media. The CM were collected after 48 hours of transfection and used for further experiments. The amount of FGF2 secreted from the MenSCs was measured using enzyme-linked immunosorbent assays (ELISAs).

### Enzyme-linked immunosorbent assay

MenSCs were seeded at 5 × 10^5^ cells/well in six-well plates. Complete medium replacement was performed when the cells reached 80% confluence. After 48 h, the cells were harvested and spun down in a centrifuge, and the supernatant was collected to measure the amount of FGF2 that was secreted according to the manufacturer’s protocols (Ameko, China). Optical densities (OD) were measured at 450 nm using an ELISA plate reader (Bio-Rad, USA).

### Statistical analysis

All analyses were performed using SPSS statistical software (version 15.0; SPSS Inc., Chicago, IL, USA). The data are expressed as the mean ± SEM from at least three independent experiments. The significance of differences between groups was assessed using one-way analysis of variance (ANOVA), and *P* < 0.05 was considered to indicate statistical significance.

## Results

### Identification and characterization of MenSCs

**Fig. 1:**
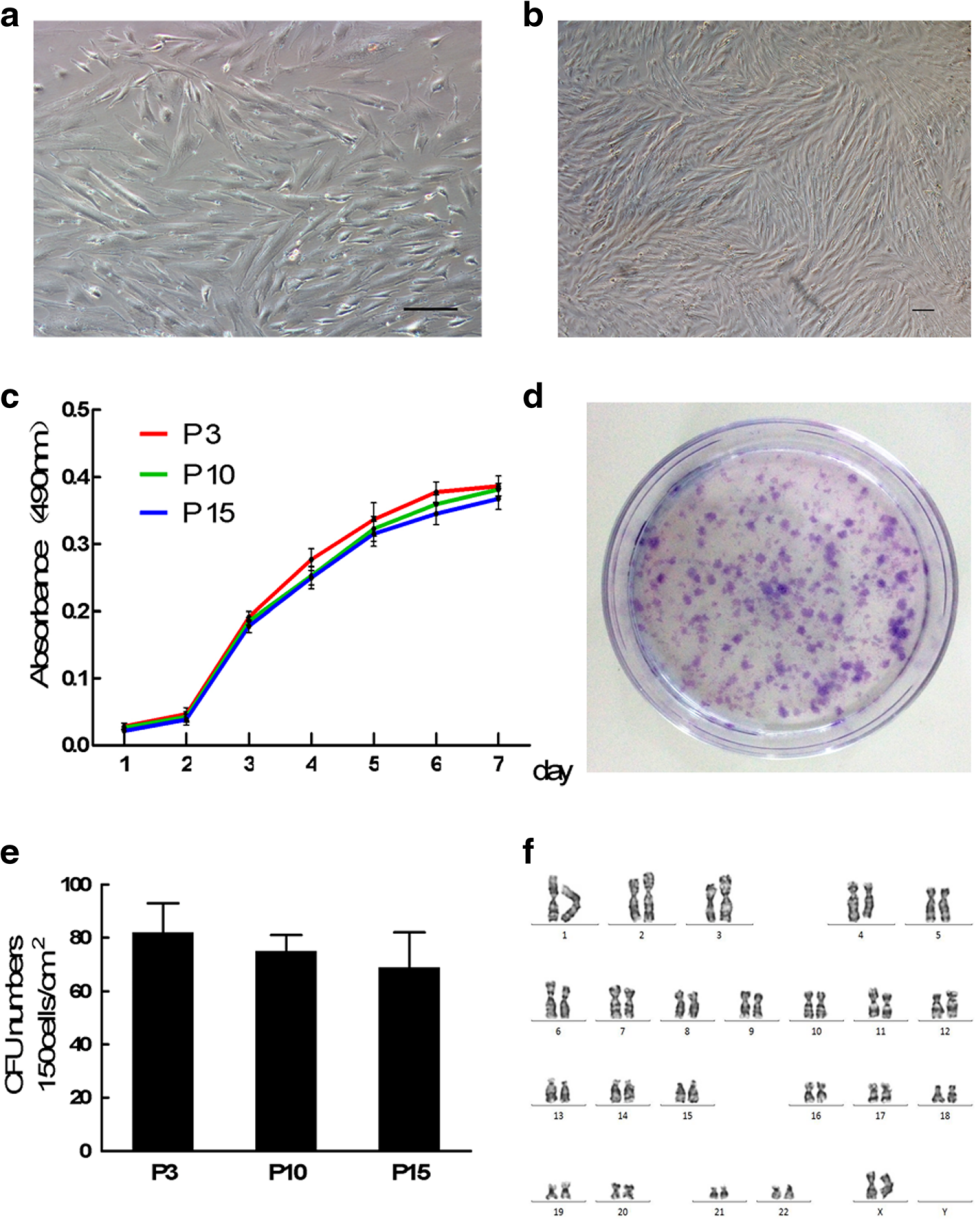
Proliferation and colony-forming ability of MenSCs. **a** Morphology of primary MenSCs: passage 0. *Scale bar* = 100 μm. **b** Morphology of passage cells: passage 3 (*P3*). *Scale bar* = 100 μm. **c** Growth curves for MenSCs were assessed using MTT assays. **d** Culture dish displaying the distribution of colonies. **e** The number of colony-forming units (*CFU*) was counted during P3, passage 10 (*P10*), and passage 15 (*P15*). **f** Cytogenetic analysis of MenSCs at passage 25

The isolated MenSCs exhibited a fibroblast-like spindle morphology (Fig. 1a, b) and maintained stable proliferation capacities. We found that the cells reached the logarithmic phase within 2–6 days, while the stagnate phase began on day 6 (Fig. 1c). To study the ability of MenSCs to self-renew, the cells were dissociated to form single cell suspensions and plated at low density (150 cells/cm^2^). Figure 1d shows a typical cloning dish containing colonies of varying sizes after 14 days of culture in vitro. To assess the stability of MenSC colony-forming ability, the cells were seeded at different passages (P3, P10, and P15). We found that there was no significant change in colony numbers between early and late passages (Fig. 1e). Additionally, we analysed the karyotypes of MenSCs. Even at passage 25, the cells continued to display a normal karyotype (Fig. 1f).

**Fig. 2:**
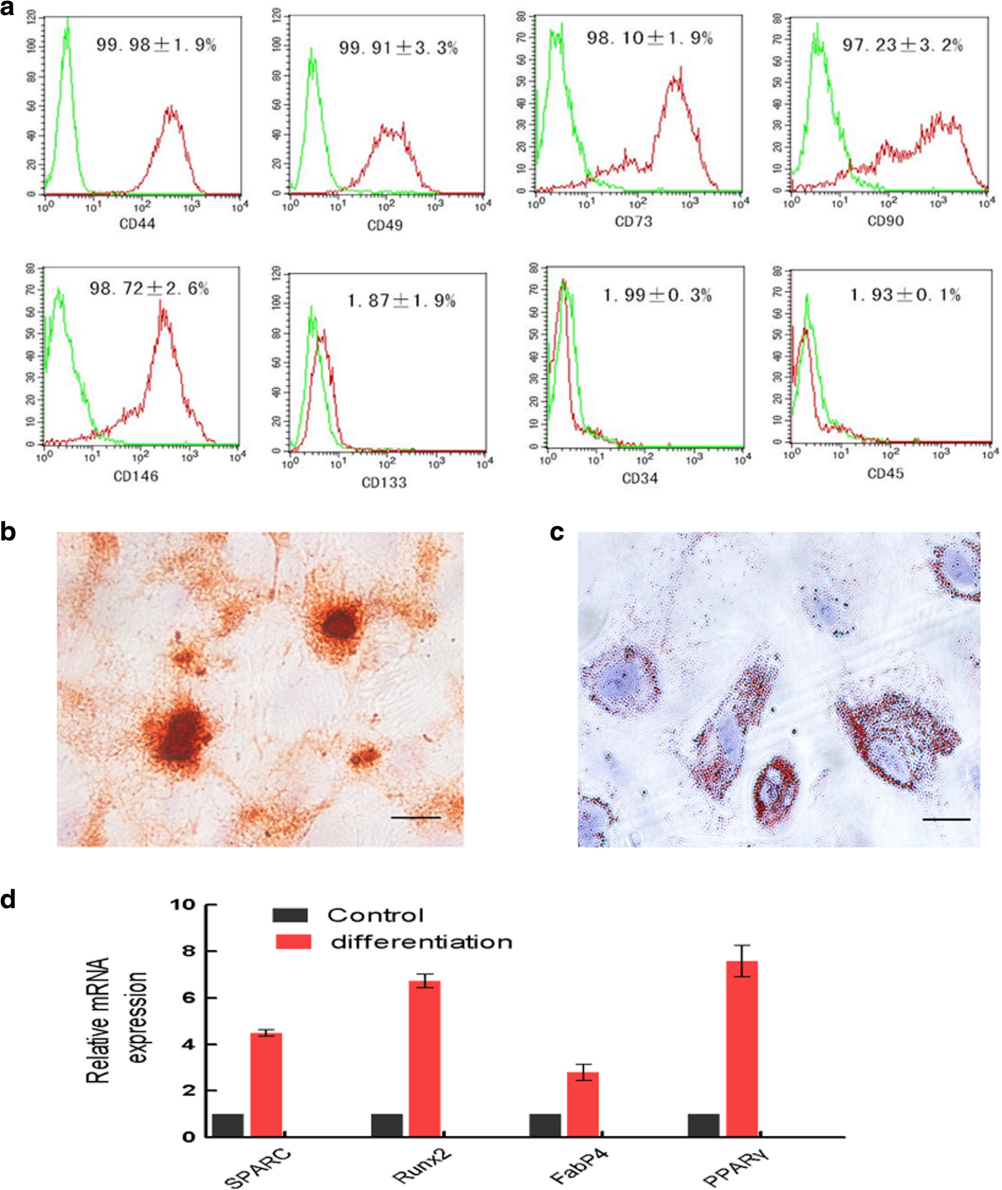
MenSCs express high levels of mesodermal antigens and display multi-lineage capacities. **a** Flow cytometry histograms showing the expression level of the indicated cell surface markers on MenSCs. **b** Multipotent differentiation of MenSCs in vitro: osteogenic differentiation is indicated by Alizarin Red reactivity. *Scale bar* = 100 μm. **c** Multipotent differentiation of MenSCs in vitro: adipogenic differentiation was visualized using Oil Red staining, which reveals lipid droplets. *Scale bar* = 100 μm. **d** Differentiated MenSCs were identified using qPCR analysis to evaluate that expression of osteogenic (*SPARC* and *Runx2*) and adipogenic (*FabP4* and *PPARγ*) markers. Expression levels of each gene were compared with the levels in undifferentiated MenSCs (data shown as mean ± SEM)

Flow cytometry was used to identify the surface markers expressed by MenSCs at P3 (Fig. 2a). MenSCs expressed surface markers similar to those expressed by MSCs. These included CD90 (97.23 ± 3.2%), CD44 (99.98 ± 1.9%), CD146 (98.72 ± 2.6%), CD49 (99.91 ± 3.3%), and CD73 (98.10 ± 1.9%). MenSCs did not express haematopoietic cell surface markers, such as CD34 (1.99 ± 0.3%), CD45 (1.93 ± 0.1%), and CD133 (1.87 ± 1.9%).

To determine the multi-differentiation potential of MenSCs, we cultured cells at P3 in osteogenenic or adipogenic differentiation-inducing culture medium. As a result, the cells successfully differentiated into osteoblasts or adipocytes, as shown by histochemical staining (Fig. 2b, c), and expressed the correct tissue-specific markers, as shown by qPCR (Fig. 2d). Mineral deposits were clearly visualized when Alizarin Red staining was used to analyse osteogenic differentiated cells. Furthermore, bone-associated genes, including *SPARC* and *Runx2*, were up-regulated in these cells. In adipogenic differentiated cells, lipid droplets were observed to have accumulated when the cells were stained using Oil Red O, and the *PPARγ* and *FabP4* genes were up-regulated.

### Treatment with MenSCs improves ovarian function in POF mice

**Fig. 3:**
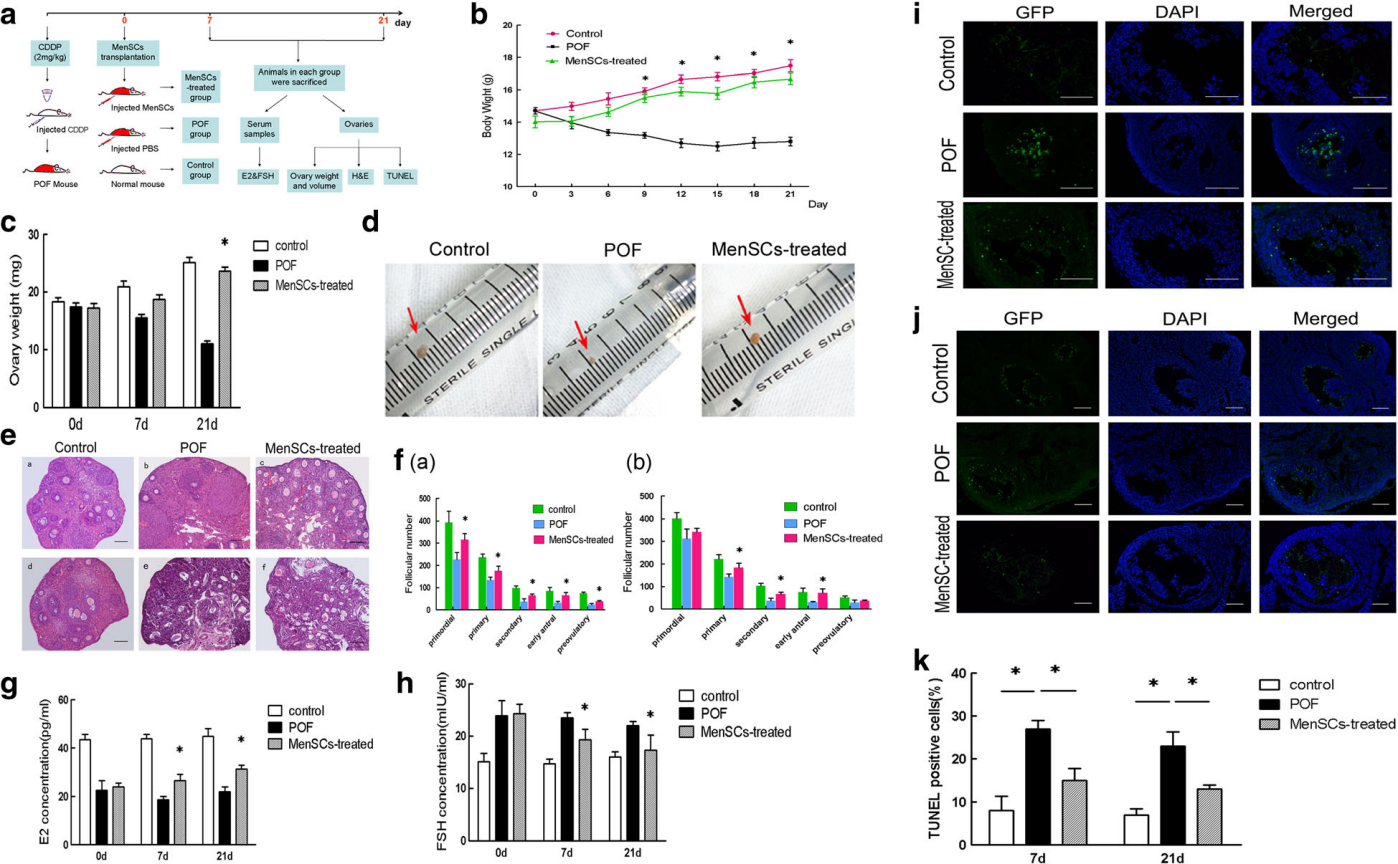
MenSC transplantation improves ovarian function after chemotherapy-induced injury. **a** Schematic of the experimental procedure used to explore the reparative effects of MenSCs in POF mice. **b** Changes in body weight between three groups (data expressed as mean ± SEM, *P < 0.05). **c** Changes in ovary weight across the three groups after 7 and 21 days (data expressed as mean ± SEM, *P < 0.05). **d** Macroscopic ovarian sizes in the three groups after 21 days. **e** Representative images showing H&E-stained ovary tissue sections in each group after 7 and 21 days. *Scale bars* = 100 μm. **f** Changes in follicle numbers in the three groups at 7 days (*a*) and 21 days (*b*) after MenSC transplantation (data expressed as mean ± SEM, *P < 0.05). **g** Serum E2 levels measured in each of the three groups. **h** Serum FSH levels measured in each of the three groups (data expressed as mean ± SEM, *P < 0.05). **i** Representative photograph showing TUNEL staining in ovary tissue sections after 7 days in each of the three groups. **j** Photograph showing TUNEL staining in ovary tissue sections after 21 days in each of the three groups. TUNEL-positive cells labelled *green*, and nuclei labelled *blue* (DAPI). *Scale bars* = 200 μm. **k** Quantitative analysis showing the percentage of TUNEL-positive cells in each group at 7 and 21 days after treatment (data expressed as mean ± SEM, *P < 0.05). *CDDP* cisplatin, *DAPI* 4′,6-diamidino-2-phenylindole, *E2* oestradiol, *FSH* follicle-stimulating hormone, *GFP* green fluorescence protein, *H&E* haematoxylin and eosin, *MenSC* menstrual-derived stem cell, *PBS* phosphate-buffered saline, *POF* premature ovarian failure, *TUNEL* terminal deoxynucleotidyl transferase mediated dUTP nick end labelling

To investigate the effects of MenSC transplantation on ovarian function, we recorded changes across the three groups in body weight at various time points during the study period (Fig. 3a). We consequently observed that body weight was significantly higher in the MenSC-treated group than in the POF group beginning on the 9th day after cell transplantation (Fig. 3b).

We also weighed the ovaries at 7 and 21 days after MenSC transplantation and found that after 21 days the ovaries obtained from MenSC-treated mice weighed significantly more than those obtained from the mice in the POF group (Fig. 3c). In addition, Fig. 3d shows that ovary sizes were different across the three groups after 21 days.

Next, the ovaries in the three groups were collected for pathological analysis. The ovaries in the POF group were more atrophic than the ovaries in the control group, and they exhibited a clear reduction in the number of follicles, especially primordial follicles, during various stages of development (Fig. 3e).

Additionally, we quantified follicle numbers over the course of each treatment. Remarkably, MenSC transplantation substantially increased the number of healthy follicles. After 7 days, there were significantly more primordial follicles (316 ± 15.59), primary follicles (176 ± 12.12), secondary follicles (64 ± 4.04), early antral follicles (65 ± 7.51), and pre-ovulatory follicles (38 ± 2.31) in the MenSC-treated group than in the POF group (Fig. 3f *a*). After 21 days, there were significantly more primary follicles (184 ± 10.97), secondary follicles (66 ± 4.62), and early antral follicles (71 ± 4.62) in the MenSC-treated group than in the POF group, and more pre-ovulatory follicles (36 ± 2.31) and primordial follicles (342 ± 9.81) than in the POF group but these differences were not statistically significant (Fig. 3f *b*).

Serum sex hormone levels were significantly different between the MenSC-treated group and the POF group. At 7 and 21 days, the level of E2 was significantly higher, while the level of FSH was lower in the group treated with MenSCs (Fig. 3g, h).

To further explore the protective effects conferred by MenSCs against the chemotherapy-induced loss of follicles, we conducted TUNEL staining at 7 and 21 days after cell infusion and then quantified the number of apoptotic nuclei per total number of nuclei in each section. Interestingly, TUNEL-positive GCs were clearly observed in the POF group, and the results suggested that apoptosis was largely restricted to the GC layer of follicles, in close proximity to the oocytes. In contrast, MenSC transplantation decreased the number of TUNEL-positive cells (Fig. 3i, j), indicating that the MenSCs reduced injury to the ovary by inhibiting apoptosis. Figure 3k shows the percentage of TUNEL-positive cells that were observed in each experimental group.

### In-vivo MenSC tracking

**Fig. 4:**
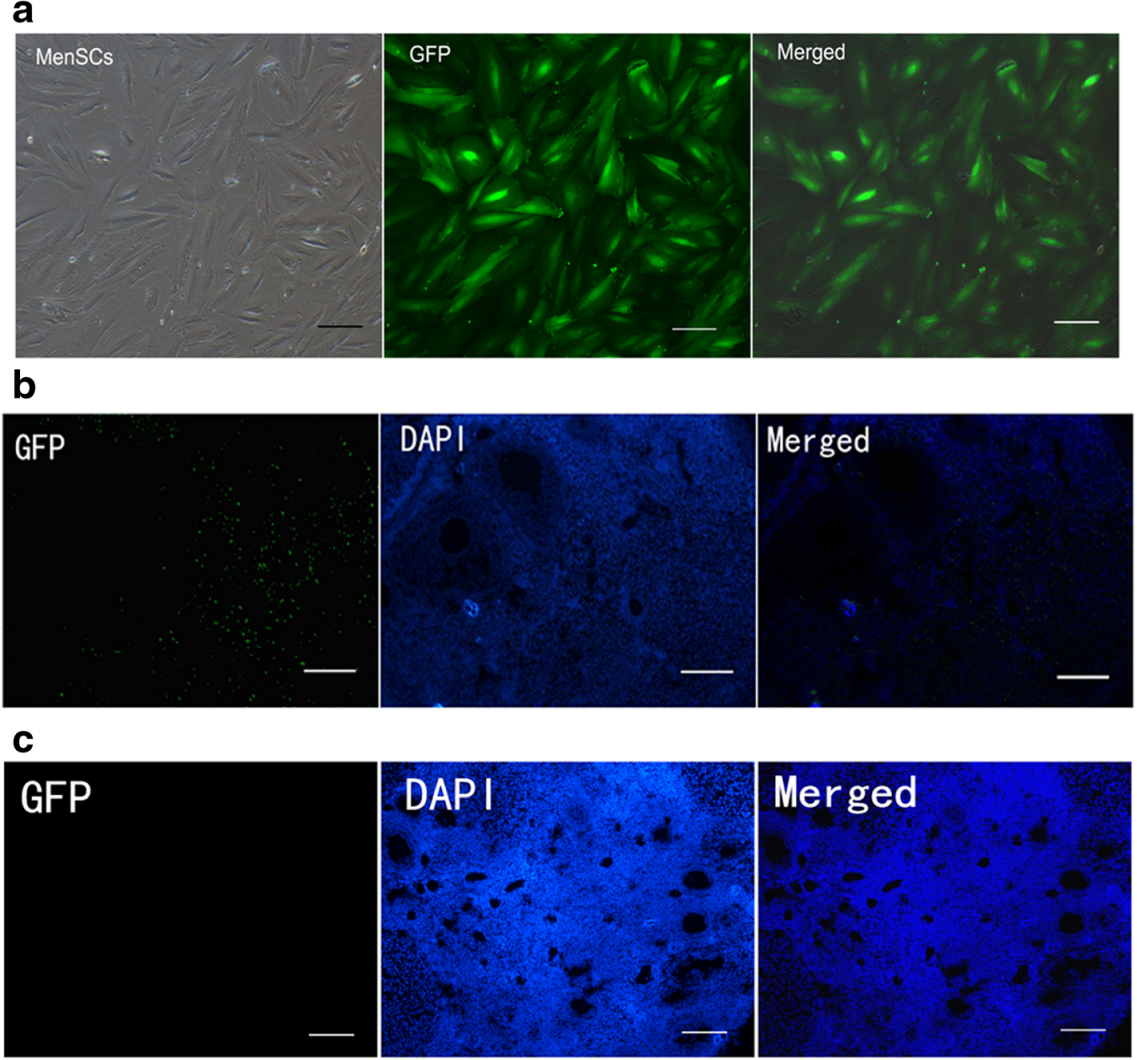
In-vivo MenSC tracking. **a** GFP-MenSCs efficiently expressed GFP in vitro. **b** Transplanted cells were observed at 7 days after infusion, and almost all of these cells were located in the ovarian interstitium and not in follicles. *Scale bars* = 100 μm. **c** No fluorescent signals in the ovaries at 21 days after cell transplantation. *Scale bars* = 100 μm. *DAPI* 4′,6-diamidino-2-phenylindole, *GFP* green fluorescence protein

As already shown, MenSCs affect the extent of ovary injury. These cells may be capable of migrating towards to the ovary. We traced the fate of injected MenSCs by injecting mice with GFP-MenSCs. Both ovaries were subsequently harvested from each mouse, and frozen sections were prepared and analysed to detect the number and location of labelled cells. MenSCs transfected with a lentivirus expressing the GFP gene efficiently and stably expressed GFP efficiently in vitro (Fig. 4a). In the mice, we found that GFP-positive cells were located in the interstitium but not in follicles at 7 days after infusion (Fig. 4b), and at 21 days there were no fluorescent signals in the ovaries (Fig. 4c).

### Effects of MenSC-derived CM on POF mice in vivo

**Fig. 5:**
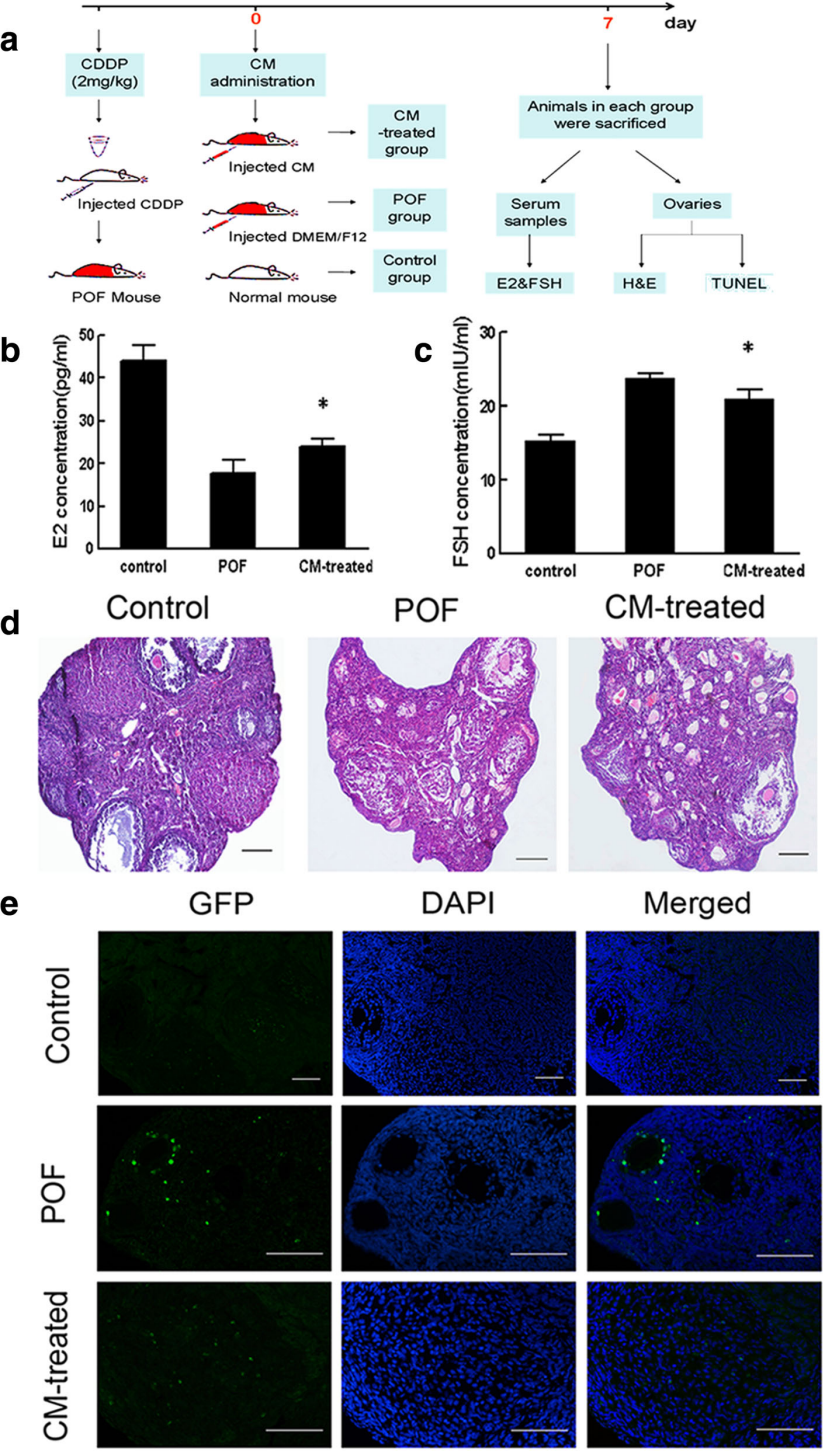
CM obtained from MenSCs improve ovarian function following chemotherapy-induced injury. **a** Schematic of the experimental procedure used to explore the reparative effects of CM in POF mice. **b** Serum E2 levels were measured in each of the three groups after 7 days. **c** Serum FSH levels were measured in each of the three groups after 7 days (data expressed as mean ± SEM, *P < 0.05). **d** Representative photomicrograph showing the results of H&E staining in each group at 7 days after injury. *Scale bars* = 100 μm. **e** Apoptosis evaluated using TUNEL staining in each group. *Scale bars* = 200 μm. *CM* conditioned media, *CDDP* cisplatin, *DAPI* 4′,6-diamidino-2-phenylindole, *E2* oestradiol, *FSH* follicle-stimulating hormone, *GFP* green fluorescence protein, *H&E* haematoxylin and eosin, *POF* premature ovarian failure, *TUNEL* terminal deoxynucleotidyl transferase mediated dUTP nick end labelling

As already shown, GFP-positive MenSCs were located in the interstitium, indicating that MenSCs may not differentiate to oocytes directly but that they may improve ovarian function via other mechanisms. Previous studies have demonstrated that MenSCs secrete a variety of cytokines that could promote the growth and renewal of the human endometrium [36]. To investigate whether MenSCs participate in ovarian recovery via paracrine activity, we collected CM from cultures of MenSCs, and then concentrated and injected them into mice (CM-treated group). The control group was injected with an equal volume of DMEM/F12 instead (Fig. 5a). Remarkably, at 7 days after injection, the levels of E2 were significantly higher and FSH levels were significantly lower in the CM-treated group than in the POF group (Fig. 5b and c, respectively). In addition, a study of tissue pathology revealed that ovarian functions were improved and adverse effects were ameliorated after CM administration: the ovarian fibrosis was reduced (Fig. 5d). Moreover, the rate of GC apoptosis was lower in the CM-treated group than in the POF group (Fig. 5e).

### FGF2 participates in ovarian injury repairs

**Fig. 6:**
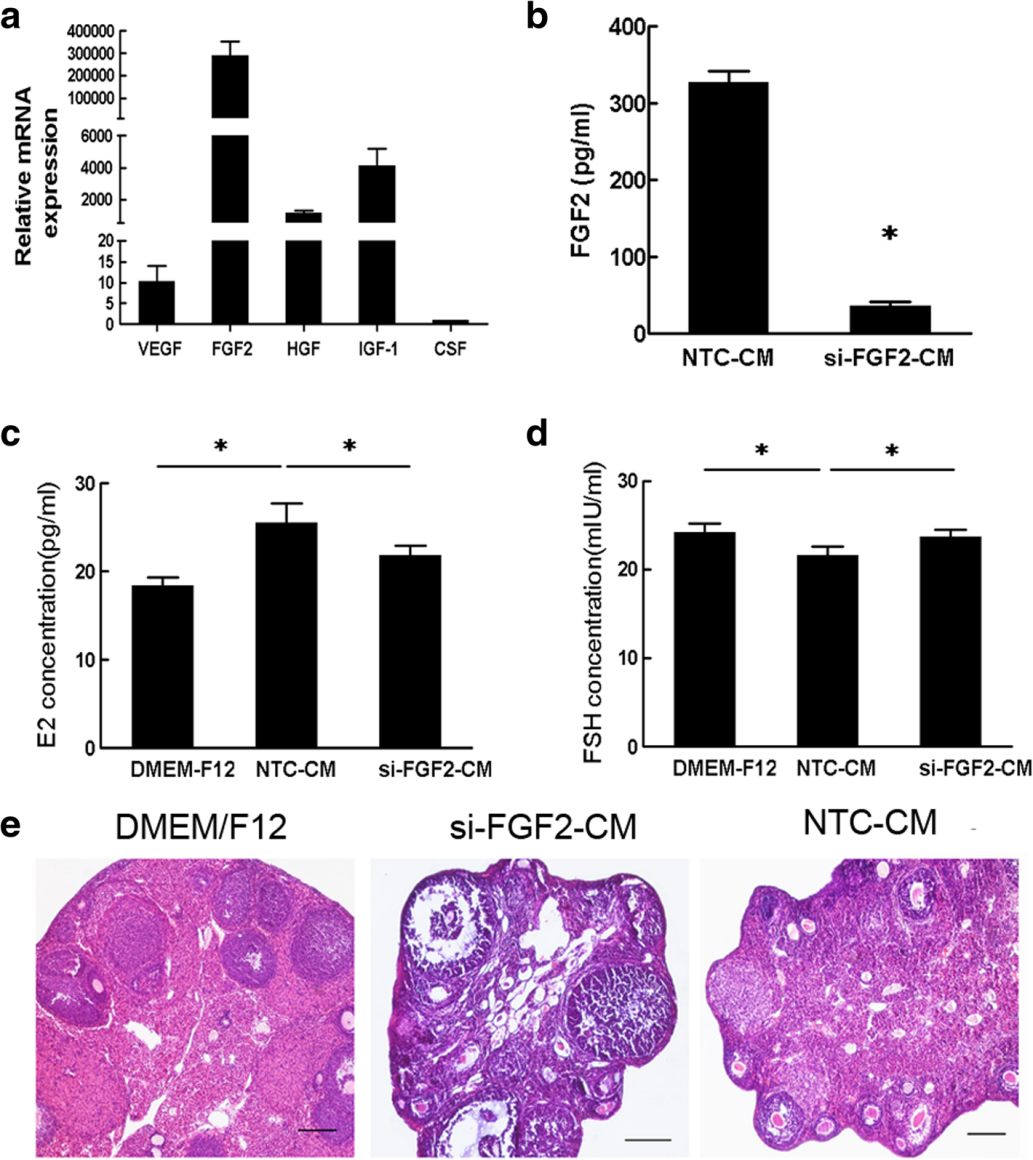
Restorative effects of MenSCs in POF are mediated via FGF2. **a** Relative mRNA expression levels of cytokines in MenSCs. **b** Cells transfected with the FGF2 siRNA expressed significantly lower levels of FGF2, as shown by ELISA (data expressed as mean ± SEM, *P < 0.05). **c** Comparison of serum levels of E2 between DMEM/F12-treated, NCT-CM-treated, and si-FGF2-CM-treated mice after 7 days. **d** Comparison of FSH levels in DMEM/F12-treated, NTC-CM-treated, and si-FGF2-CM-treated mice after 7 days (data expressed as mean ± SEM, *P < 0.05). **e** Representative images showing H&E staining after 7 days. Lesions were more pronounced in the ovaries of si-FGF2-CM-injected mice than in mice treated with NTC-CM. *Scale bars*: 100 μm. *CM* conditioned media, *E2* oestradiol, *FGF2* fibroblast growth factor 2, *FSH* follicle-stimulating hormone, *NTC* negative transfection control siRNAs, *IGF-1*insulin-like growth factor-1, *VEGF* vascular endothelial growth factor, *HGF* hepatocyte growth factor), *FGF2*fibroblast growth factor 2, *CSF* colony stimulating factor

To gain a further insight into the potential ovary-reparative mechanisms underlying the functional benefits observed following treatment with CM obtained from MenSCs, we evaluated the levels of cytokines and growth factors known to be associated with ovary reparative functions. These included insulin-like growth factor-1 (IGF-1), vascular endothelial growth factor (VEGF), hepatocyte growth factor (HGF), fibroblast growth factor 2 (FGF2), and granulocyte-colony stimulating factor (G-CSF). We determined the level of each markers using qPCR. Interestingly, we found that MenSCs expressed extremely high levels of FGF2 (Fig. 6a).

To further explore the potential ovary-reparative role of FGF2 derived from MenSCs, a siRNA was used to disrupt the expression of FGF2. FGF2 was efficiently knocked down at the protein expression level, as shown by ELISA, by the siRNA but not by the NTC (Fig. 6b).

When FGF2 expression was knocked down by the siRNA, the CM obtained from MenSCs no longer significantly increased serum levels of E2 or decreased FSH levels (Fig. 6c, d). Moreover, the reparative effect of the CM obtained from MenSCs on ovarian injury was also substantially weakened: there were fewer follicles and there was more ovarian interstitial fibrosis in the siRNA-treated group than in the NTC group (Fig. 6e). These data suggest that the therapeutic effect of MenSCs is partially mediated by the secretion of FGF2.

## Discussion

In the present study, we demonstrated that MenSCs isolated from menstrual blood exhibit the properties of MSCs. We explored the potential effects of MenSCs on chemotherapy-induced ovarian damage and found that MenSCs were able to repair ovarian injury successfully.

Previous studies have shown that MSCs can be isolated from many tissues, including skin [37], adipose tissue [10], human amniotic fluid [38], the umbilical cord [39], and the heart [40]. MSCs have become an excellent source of seed cells that can be used for regenerative therapies in a variety of diseases because they have strong self-renewal and multi-differentiation capacities. However, a major drawback of these seed cells is that they were difficult to obtain or the available quantity of them is limited, which restrict their practical applications. Here, we isolated MenSCs from menstrual blood, and we show that they possess the properties of MSCs. Importantly, the most advantageous characteristic of MenSCs, aside from their accessibility and the need for only non-invasive isolation procedures to obtain them, is the possibility that cells can periodically be collected from the same donor. Additionally, as a result of the low immunogenicity and the immunoregulatory function of MenSCs, the allotransplantation of MenSCs has successfully been applied to treatments for multiple sclerosis, myocardial infarction and Parkinson’s disease [32, 41, 42]. The allotransplantation of MenSCs in patients with POF can therefore also reasonably be assumed to achieve success.

Higher therapeutic doses of low-passaged MenSCs with the same genetic background can therefore be obtained from each individual. Moreover, the low immunogenicity of MenSCs suggests that they can be used safely in allotransplantations.

However, as a prominent source of tissues that can be used in cell therapy, MenSCs also possess a broader array of functional properties that might underlie their efficacy for specific therapeutic applications. In this study, we focused on evaluating the cellular and molecular characteristics of MenSCs. The results demonstrate that MenSCs feature characteristics similar to MSCs, including an adherent spindle shape, similar surface marker expression, and multi-lineage differentiation capabilities. We have consequently focused our investigation on examining the reparative effects of MenSCs.

We developed a POF mouse model using cisplatin-induced ovarian injury, which has been shown to cause ovarian failure in humans [1, 43]. Moreover, cisplatin induces apoptosis in GCs [44], which are required for oocyte survival and follicle development [40, 45]. After 7 days, mice that were intraperitoneally injected with cisplatin lost a significant amount of body and ovary weight. Histological analyses indicated that the numbers of follicles in various stages of development were clearly reduced. This was especially true for primordial follicles, which stopped developing and exhibited dysmaturity. Fibrosis was visible in the ovarian stroma, similar to the pathological changes that are observed in clinical cases after treatment with chemotherapy [46]. However, after MenSC transplantation, ovarian function significantly improved: fibrosis was ameliorated, the number of follicles was increased, apoptosis was decreased in GCs, and hormone levels were normalized. Interestingly, we found that while apoptosis was largely restricted to the GC layer of follicles after POF injury, GFP-MenSCs localized to the ovarian stroma rather than follicles, indicating that MenSCs might migrate to the injured areas of the ovary and repair tissues by improving and promoting the regeneration of resident cells. This activity is most probably mediated by paracrine effects that are initiated in response to MenSC-secreted cytokines and growth factors that prevent necrosis and programmed cell death. These cytokines and growth factors may also be instrumental in promoting cell proliferation. We therefore propose that the tissue salvage observed in the MenSC-treated mice included a component of endogenous regeneration as well as ovarian protection, and that these activities were mainly and perhaps exclusively the result of paracrine activity. Previous studies have demonstrated that MSCs express distinct cytokines which enhance cell survival, proliferation, and function and thereby exert a positive effect on repairing tissue damage [47–49]. To research the reparative mechanism underlying the effects of MenSCs, we tested the effects of CM obtained from MenSCs. The results of these experiments confirmed that the MenSC-derived CM played a cytoprotective role and had anti-apoptotic properties. We investigated the expression levels of a variety of cytokines known to be secreted by MenSCs and surprisingly found that these cells produced much higher levels of FGF2 than other cytokines. Among bioactive factors, FGF2 is known to be essential for angiogenesis and for the proliferation and remodelling of endometrial cells, and it has also been shown to play important roles in repairing and regenerative damaged tissues [50–52]. The concentrated CM that were injected into the mice contained an abundance of FGF2, which effectively repaired ovarian function by decreasing the fibrosis in ovarian interstitium and promoting the growth of follicles. These effects consequently improved the secretion of E2. In spite of the fact that the expression levels of IGF-1 and HGF were slightly higher in MenSCs, when FGF2 expression was knocked using a siRNA the reparative activity of the MenSCs was significantly abrogated. These results further support the notion that the therapeutic effects of MenSCs are likely to be partially mediated by the secretion of FGF2.

## Conclusions

Our research shows that MenSCs can repair ovarian injury, stimulate regeneration, and improve ovarian function. Furthermore, the reparative effects of these cells were mainly based on their paracrine mechanism by secreting FGF2. MenSC transplantation may provide an effective and novel method for treating POF.

## Abbreviations

CDDP: Cisplatin
CFU: Colony-forming unit
FGF2: Fibroblast growth factor 2
G-CSF: Granulocyte-colony stimulating factor
HGF: Hepatocyte growth factor
HRT: Hormone replacement therapy
IGF-1: Insulin-like growth factor-1
MenSC: Menstrual-derived stem cell
VEGF: vascular endothelial growth factor
