# Erratum to: Study of the reparative effects of menstrual-derived stem cells on premature ovarian failure in mice

**DOI:** 10.1186/s13287-017-0526-1

**Published:** 2017-03-08

**Authors:** Zhen Wang, Yueling Wang, Ting Yang, Jing Li, Xinyuan Yang

**Affiliations:** 1 Department of Gynecology and Obstetrics, First Affiliated Hospital, Xi’an Jiaotong University, Xi’an, 710061 People’s Republic of China; 2 Center for Translational Medicine, First Affiliated Hospital of Xi’an Jiaotong University, Xi’an, 710061 People’s Republic of China

## Erratum

The original article [1] contains errors in Figs. 3i, j and 5e whereby the first column of each sub-panel is incorrectly labelled as having used GFP-staining; instead, the images were generated using TUNEL assays.

**Fig. 3:**
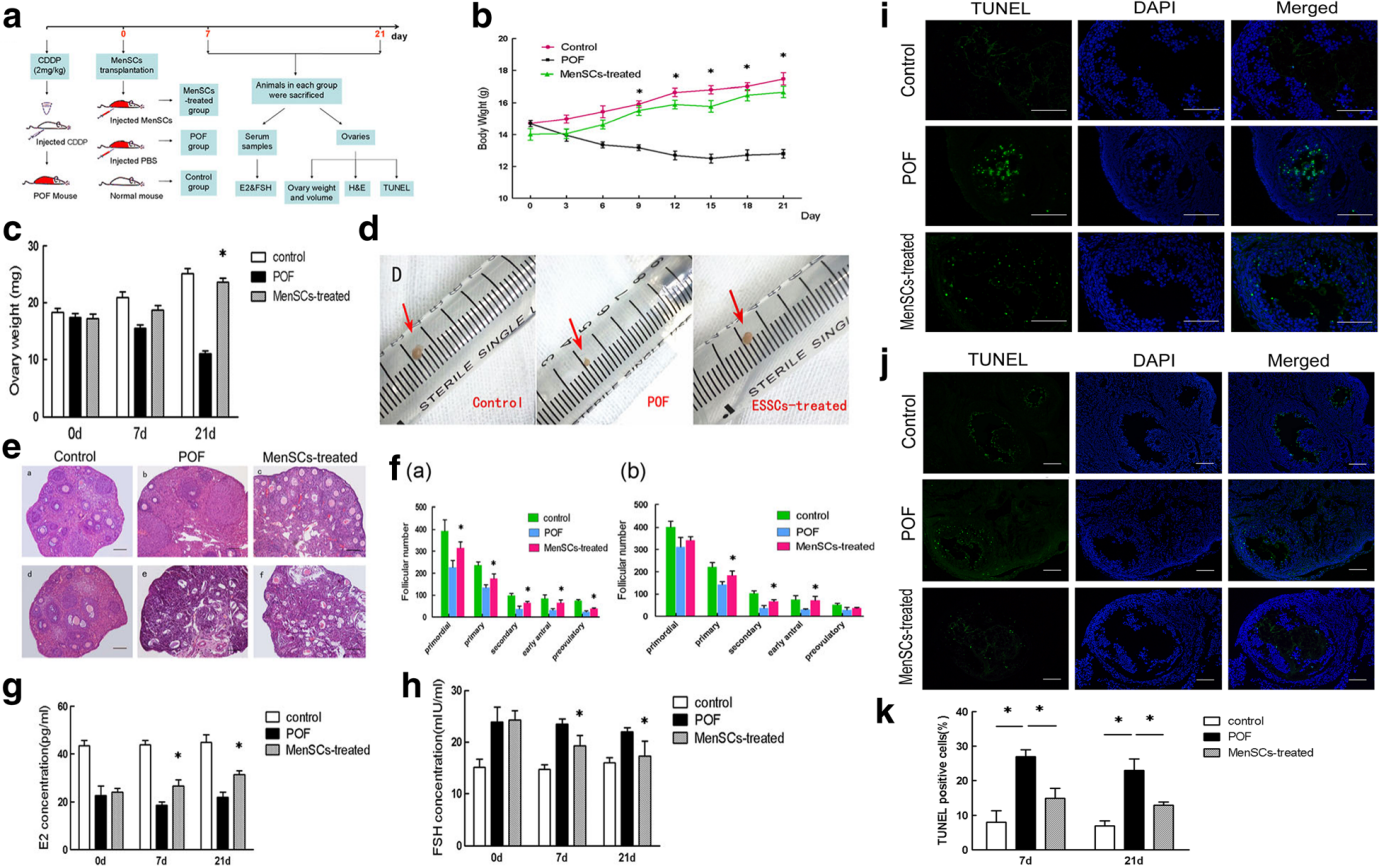
MenSC transplantation improves ovarian function after chemotherapy-induced injury. **a** Schematic of the experimental procedure used to explore the reparative effects of MenSCs in POF mice. **b** Changes in body weight between three groups (data expressed as mean ± SEM, *P < 0.05). **c** Changes in ovary weight across the three groups after 7 and 21 days (data expressed as mean ± SEM, *P < 0.05). **d** Macroscopic ovarian ovarian sizes in the three groups after 21 days. **e** Representative images showing H & E-stained ovary tissue sections in each group after 7 and 21 days. *Scale bars* = 100 μm. **f** Changes in follicle numbers in the three groups at 7 days (*a*) and 21 days (*b*) after MenSC transplantation (data expressed as mean ± SEM, *P < 0.05). **g** Serum E2 levels measured in each of the three groups. **h** Serum FSH levels measured in each of the three groups (data expressed as mean ± SEM, *P < 0.05). **i** Representative photograph showing TUNEL staining in ovary tissue sections after 7 days in each of the three groups. **j** Photograph showing TUNEL staining in ovary tissue sections after 21 days in each of the three groups. TUNEL-positive cells labelled *green*, and nuclei labelled *blue* (DAPI). *Scale bars* = 200 μm. **k** Quantitative analysis showing the percentage of TUNEL-positive cells in each group at 7 and 21 days after treatment (data expressed as mean ± SEM, *P < 0.05). *CDDP* cisplatin, *DAPI* 4′,6-diamidino-2-phenylindole, *E2* oestradiol, *FSH* follicle-stimulating hormone, *H&E* haematoxylin and eosin, *MenSC* menstrual-derived stem cell, *PBS* phosphate-buffered saline, *POF* premature ovarian failure, *TUNEL* terminal deoxynucleotidyl transferase mediated dUTP nick end labelling

**Fig. 5:**
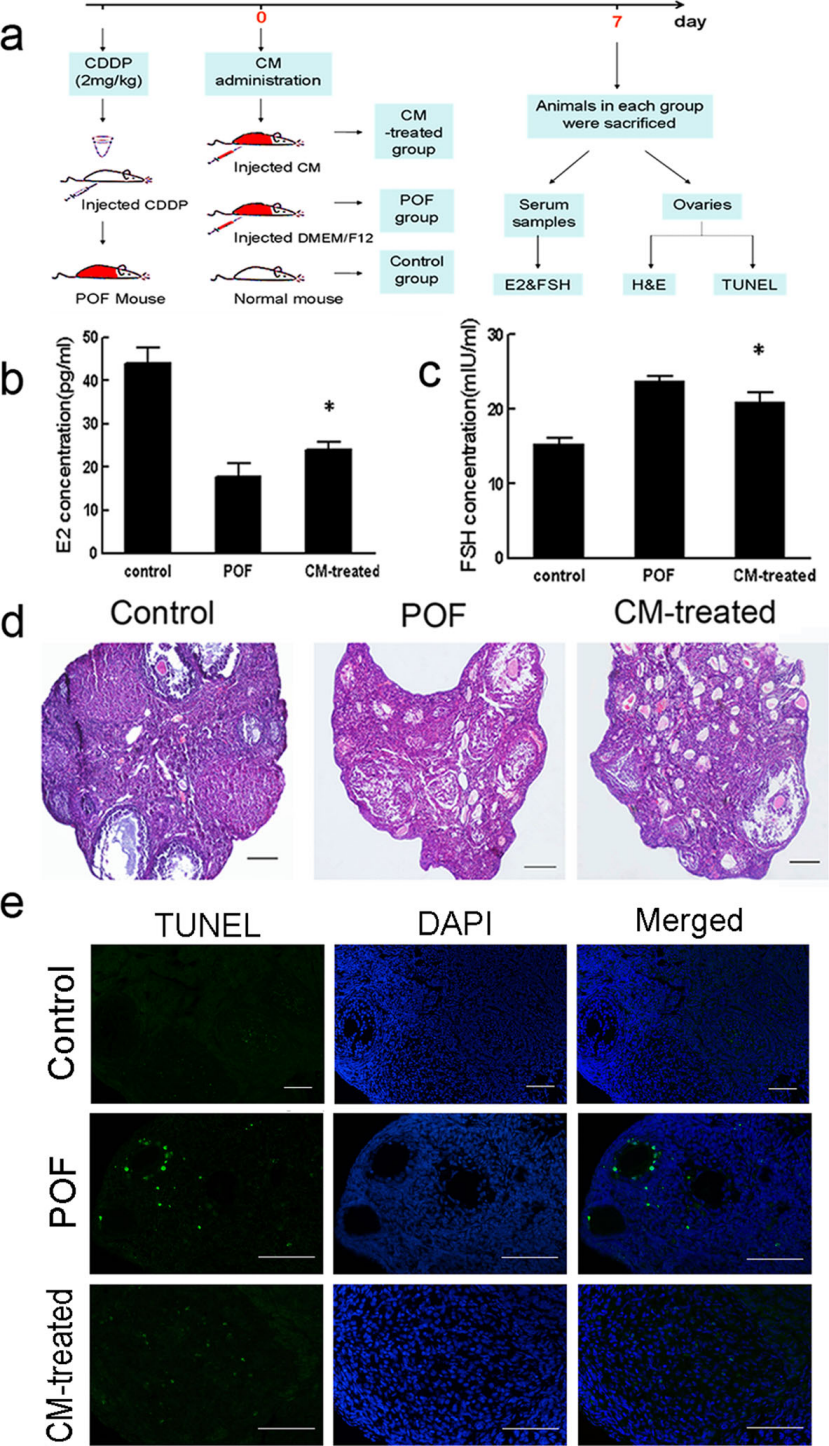
CM obtained from MenSCs improve ovarian function following chemotherapy-induced injury. **a** Schematic of the experimental procedure used to explore the reparative effects of CM in POF mice. **b** Serum E2 levels were measured in each of the three groups after 7 days. **c** Serum FSH levels were measured in each of the three groups after 7 days (data expressed as mean ± SEM, *P < 0.05). **d** Representative photomicrograph showing the results of H&E staining in each group at 7 days after injury. *Scale bars* = 100 μm. **e** Apoptosis evaluated using TUNEL staining in each group. *Scale bars* = 200 μm. *CM* conditioned media, *CDDP* cisplatin, *DAPI* 4′,6-diamidino-2-phenylindole, *E2* oestradiol, *FSH* follicle-stimulating hormone, *H&E* haematoxylin and eosin, *POF* premature ovarian failure, *TUNEL* terminal deoxynucleotidyl transferase mediated dUTP nick end labelling

Consequently, the correct version of each figure can be seen below in this erratum.
